# A case of prenatal diagnosis of 18p deletion syndrome following noninvasive prenatal testing

**DOI:** 10.1186/s13039-019-0464-y

**Published:** 2019-12-21

**Authors:** Ganye Zhao, Peng Dai, Shanshan Gao, Xuechao Zhao, Conghui Wang, Lina Liu, Xiangdong Kong

**Affiliations:** Genetics and Prenatal Diagnosis Center, The First Affiliated Hospital of Zhengzhou University, Henan Engineering Research Center for Gene Editing of Human Genetic Disease, Erqi District, Zhengzhou, China

**Keywords:** NIPT, 18p deletion syndrome, Karyotype, CNV-seq, Prenatal diagnosis

## Abstract

**Background:**

Chromosome 18p deletion syndrome is a disease caused by the complete or partial deletion of the short arm of chromosome 18, there were few cases reported about the prenatal diagnosis of 18p deletion syndrome. Noninvasive prenatal testing (NIPT) is widely used in the screening of common fetal chromosome aneuploidy. However, the segmental deletions and duplications should also be concerned. Except that some cases had increased nuchal translucency or holoprosencephaly, most of the fetal phenotype of 18p deletion syndrome may not be evident during the pregnancy, 18p deletion syndrome was always accidentally discovered during the prenatal examination.

**Case presentations:**

In our case, we found a pure partial monosomy 18p deletion during the confirmation of the result of NIPT by copy number variation sequencing (CNV-Seq). The result of NIPT suggested that there was a partial or complete deletion of X chromosome. The amniotic fluid karyotype was normal, but result of CNV-Seq indicated a 7.56 Mb deletion on the short arm of chromosome 18 but not in the couple, which means the deletion was de novo deletion. Finally, the parents chose to terminate the pregnancy.

**Conclusions:**

To our knowledge, this is the first case of prenatal diagnosis of 18p deletion syndrome following NIPT.NIPT combined with ultrasound may be a relatively efficient method to screen chromosome microdeletions especially for the 18p deletion syndrome.

## Background

Noninvasive prenatal testing (NIPT) is widely used in the screening of common fetal chromosome aneuploidy including trisomy 21, trisomy 18 and trisomy 13, due to its high sensitivity and specificity [1, 2]. However, the common trisomies comprises only approximately 75% of aneuploidies [3], about 24% of the reported anomalies would have been missed [4]. Other rare aneuploidies and segmental deletions and duplications should also be concerned. As NIPT is based on the low-coverage whole genome sequencing of maternal plasma cell-free DNA, it can detect all chromosomes actually. Subchromosomal deletions and duplications would also be detected by NIPT [5].

Chromosome 18p deletion syndrome, a disease caused by the complete or partial deletion of the short arm of chromosome 18, was first reported by Groucy and colleagues in 1963, with an incidence of about 1/50000 in live births [6]. Lack of 18p loss syndrome according to the location and size eventually led to the large difference of clinical features. The main symptoms may involve short stature, low intelligence, special features, language development backwardness, low muscle tone, brain malformation, skeletal deformities, reproductive system dysplasia, kidney or abnormal cardiac birth defects, such as skin hair and serum immunoglobulin A absent or reduced symptoms [7].

There is no specific treatment for the syndrome. The prenatal diagnosis of 18p deletion syndrome is significant for early management and prevention. As the clinical manifestations of the fetus during the pregnancy vary widely. Majority cases were accidentally diagnosed [8]. The prenatal diagnosis of the syndrome still presents as a challenge because of its untypical clinical presentation [9].

Recently, we found a case of a mid-pregnancy woman with an abnormal chromosome 18p deletion following an aberrant NIPT result. The NIPT results showed a deletion on chromosome X. Karyotype analysis and copy number variation sequencing (CNV-Seq) were then used to confirm the result of NIPT. Finally, we detected a 7.56 Mb pure deletion at 18p11.32p11.23 of the fetus.

## Case presentation

**Fig. 1:**
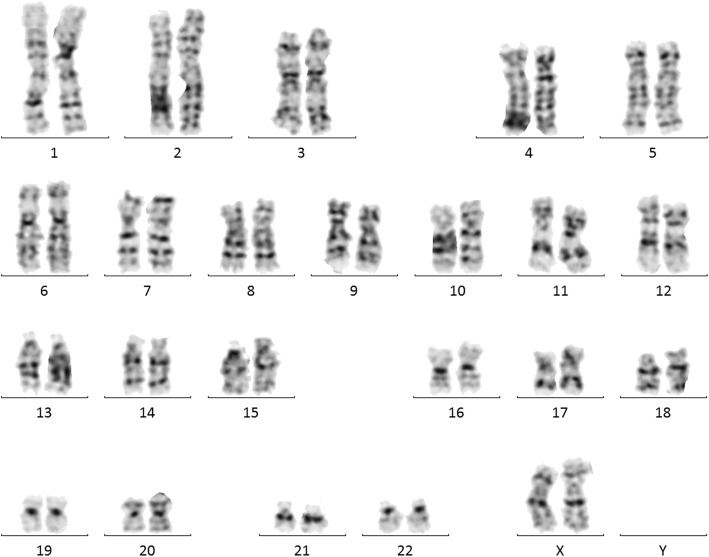
Karyotype analysis of maternal amniotic fluid showing no significant fetal chromosomal abnormalities (46, XX)

**Fig. 2:**
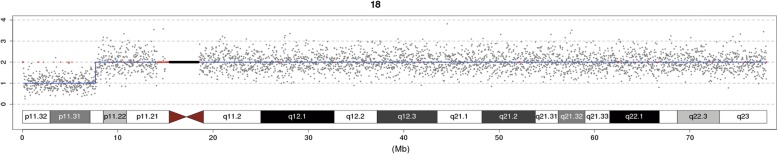
Copy number variation of maternal amniotic fluid showing that a deletion of 7.56 Mb on chromosome 18p p11.32p11.23(seq[hg19]18 p11.32p11.23 (120000–7,680,000) × 1)

**Fig. 3:**
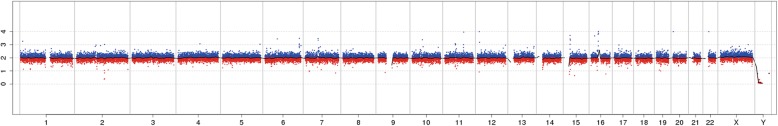
Copy number variation of the fetus’s mother was normal

**Fig. 4:**
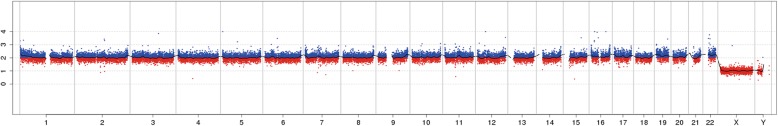
Copy number variation of the fetus’s father was normal

A 20-year-old pregnant woman with a single fetus, pregnancy 1, parturition 0, gestational age 19 weeks 1 day, was sent to Genetic and Prenatal Diagnostic Center, The First Affiliated Hospital of Zhengzhou University. The woman was 160 cm tall and weighed 70 kg. The course of her pregnancy was uneventful. Her husband was 25 years old. The couple was both healthy and not consanguineous. The ultrasound findings were normal during the whole pregnancy. NIPT was selected to screen for fetal chromosomal abnormalities. The results suggested that 21-trisomy, 18-trisomy and 13-trisomy were negative, but showed fetal ChrX-, suggesting partial or complete deletion of X chromosome. Therefore, amniotic fluid was extracted by amniocentesis at 20 weeks of gestation for cell culture analysis of fetal amniotic fluid karyotype and human genome copy number variation (CNV) was detected by high-throughput sequencing. The result of amniotic fluid karyotype was normal (Fig. 1). The result of CNV-Seq test was seq[hg19]18p11.32p11.23(120000–7,680,000)× 1(Fig. 2), suggesting the heterozygosis deletion of fetus. 18p11.32p11.23 was about 7.56 Mb, which contains 24 OMIM genes. In order to further clarify the pathogenicity of the deletion of this segment, the DNA of the couple was extracted from their peripheral blood and cnv-seq test was conducted respectively. The results showed that the couple had no chromosome abnormality (Fig. 3 and Fig. 4), which means the deletion was a de novo mutation in the fetus. Considering all of the above, this deletion was pathogenic. After informing the risk of this syndrome, the pregnant women and her families decided to terminate the pregnancy.

## Results

Peripheral blood (10 ml) was collected in Streck tubes (Streck, USA) from the pregnant woman. Cell free DNA was extracted. Sequencing library preparation and sequencing were conducted according to the instruction. Sequencing was performed using a Next-Seq CN500 Sequencing System (Illumina, USA), with the single-ended 43 bp sequencing protocol. Raw reads were mapped to hg19 reference genome and the uniquely mapped reads were analyzed. We got 4.96 million raw reads and 3.2 million uniMap reads with a fetal fraction of 8.115%. Finally, noninvasive prenatal testing results gave a Z-score of − 3.91 for chromosome X and showed that there was about a deletion of chromosome X. Then amniocentesis was conducted to verify the NIPT results with karyotype analysis and CNV-seq.

The amniocentesis was performed under the guidance of ultrasound, and 20 ml of amniotic fluid was taken. The karyotype analysis of fetal amniotic fluid exfoliated cells was performed. The result of karyotype analysis amniotic fluid showed no obvious abnormalities in fetal chromosome (Fig. 1).

CNV-seq was performed according to standard procedures as previously reported [10, 11]. In short, DNA extracted from fetal amniotic fluid or uncultured peripheral blood samples was fragmented. Then, sequencing libraries constructed were sequenced on the Next-Seq CN500 platform (Illumina, USA). The results were analyzed using the previously described algorithms [11].

The CNV-Seq analysis results were seq[hg19]18p11.32p11.23(120000–7,680,000) × 1, indicating a deletion of about 7.56 Mb on chromosome 18 p11.32p11.23 (Fig. 2). CNV-Seq analysis of the chromosomes of the couple showed no obvious abnormalities (Fig. 3 and Fig. 4). The inability to detect this microdeletion with the traditional karyotype analysis might be attributable to the low resolution of G-banding.

## Discussion and conclusions

In this case, there was a heterozygosis deficiency of 7.56 Mb in 18p11.32p11.23 (120,000–7,680,000). It contains 24 OMIM genes, including *ADCYAP1, ARHGAP28, C18orf42, CETN1, CLUL1, COLEC12, DLGAP1, EMILIN2, ENOSF1, EPB41L3, L3MBTL4, LAMA1, LPIN2, MYL12B, MYOM1, NDC80, PTPRM, SMCHD1, TGIF1, THOC1, TYMS, USP14YES1* and *ZBTB14*. There are 12 dose-sensitive genes in the short arm of chromosome 18 [6], 18p11.32p11.23 contains 5 of them: *TGIF1, DLGAP1, LAMA1, SMCHD1* and *CETN1*.The mutation or absence of *TGIF1* can cause anencephaly and pituitary dysplasia. The *LAMA1* gene is involved in the development of retina, kidney and cerebellum. *SMCHD1* gene is associated with facial shoulder brachial muscular dystrophy [12]. Genes associated with autism including *DLGAP1* and *CETN1* have a great impact on fertility, especially in males. Several patients in the Decipher database were reported as overlapping deletions on 18p with our case. The patient identified as No. 333229 had a 7.03 Mb deletion at chr18:1,835,696-8,861,381 and suffered from language disorders and neurodevelopmental abnormalities. The patient identified as No.328424 had a 4.27 Mb deletion at chr18:136,226-4,409,550 and suffered from congenital microcephaly, global developmental delay and short stature. In conclusion, the fetus was more likely to develop into a 18p partial deletion syndrome in the future.

The traditional karyotype analysis did not detect the microdeletion due to its low resolution of G-banding. Thanks to the improvements of cytogenetic techniques including chromosome microarray assay (CMA) or CNV-Seq, the microdeletions and microduplications would not be omitted.

As with this case, most of patients with 18p deletion syndrome were de novo deletions [13]. Some prenatal testing including high risk of maternal serum screening, increased nuchal translucency or holoprosencephaly (HPE) may indicate the pure 18p deletion syndrome (Table 1). Manifestations of the 18p deletion syndrome vary greatly from different patients as described above, while the pregnancy and delivery were mostly normal. The ultrasound results would be normal during all the whole pregnancy period [8, 13], which means the prenatal diagnosis of this syndrome was usually an unexpected finding during amniocentesis [8].

**Table 1:** Data of the reported cases of prenatal diagnosis of pure 18p deletion syndrome

Gestational Age	Age	Husband’s Age	Methodology	Origin	Deletion	Prenatal Diagnostic Indications	Reference
17	39	42	Karyotype; FISH	de novo	46,XY.ish del(18)(p10pter)(tel18p-, dim D18Z1)	advanced maternal age	[9]
20	32	38	Karyotype; aCGH	de novo	13.87 Mb deletions from 18p11.21 to pter	Increased nuchal translucency (INT) (5.1 mm) and a 5.4 cm crown-rump length (CRL) at 12 weeks’ gestation	[8]
18	32	NA	Karyotype; aCGH	de novo	12.68 Mb deletions from 18p11.32-p11.21	Second trimester maternal serum screening blood test: a high risk of Down syndrome (1:20)	[8]
18	31	NA	NIPT; karyotype; SNP-array	NA	6.9 Mb deletions at 18p11.32p11.31 and 7.5 Mb deletions in 18p11.23p11.21	INT from a value of 3.3 mm for 4.8 cm of CRL at 11 + 4 weeks to 4.9 mm for 5.91 cm of CRL at 12 + 2 weeks of gestation	[8]
23	24	26	Karyotype; FISH; microarray	maternal	18 Mb deletion at 18q11.1-p11.32	A history of abnormal pregnancy, firstpregnancy ended in a miscarriage in the first trimester; lost the second pregnancy due to a hydatidiform mole; Sonography showed congenital foetus malformation, including fused cerebral hemispheres, dilatation of the cerebral ventricles, a single palpebral fissure and proboscis	[14]
24	29	33	Karyotype; FISH; CMA	NA	4.5 Mb pure microdeletion at 18p11.32–11.31	multiple fetal abnormalities: fetal semilobar holoprosencephaly, median cleft lip and palate, arhinia and tetralogy of Fallot	[15]
19	36	34	Karyotype; FISH; aCGH; qf-PCR	de novo	14.06 Mb deletion at 18p11.32-p11.21	advanced maternal age and sonographic findings of craniofacial abnormalities; Level II ultrasound at 19 weeks of gestation showed HPE and median facial cleft	[16]
13	35	NA	karyotype	de novo	46,XX,del(18)(p11.2)	a crown-rump length of 79 mm and an increased nuchal translucency thickness of 3.9 mm	[17]

*Note*: *aCGH* array-based comparative genomic hybridization, *FISH* fluorescent in situ hybridization, *NA* not available (absent or unrecorded), *NIPT* non-invasive prenatal testing, *CMA* chromosome microarray assay, *qf-PCR* quantitative fluorescent polymerase chain reaction, *INT* increased nuchal translucency

The positive predictive value (PPV) for detecting 45, X was 18.39 to 66.67% [18–20]. In our center, the PPV for 45, X was 16.13% (data not published), which needs to be improved. In the all cases of high risk for 45, X in our center, this was the only one case that the result of prenatal diagnosis was pathogenic but the abnormity was discordance with the result of NIPT. The cause of this discordance was not investigated further as it was difficult to get the placenta. A possible explanation may be confined placental mosaicism as fetoplacental mosaicism was a main reason that lead to false positive or false negative results of NIPT [21]. There was a possibility that the placenta has a X chromosome deletion problem, while the fetus has a 18p deletion syndrome.

There are no obvious ultrasound indications or other traditional efficient screening ways to detect the 18p deletion syndrome. NIPT is a very efficient and accurate method for the detection of chromosome aneuploidy, especially for chromosome 13, 18 and 21. Recently, further expansion of NIPT through deeper sequencing has focused on additional screening for microdeletion and microduplications, which had a very successful screening results [22–24]. Prenatal ultrasound of our case was normal, this chromosome deletion would be missed if the woman did not choose NIPT as her prenatal testing. Though, the result of NIPT was discordant with the result of prenatal diagnosis, it gave a clue for the possibility of chromosome abnormity.

Reports of the prenatal diagnosis of 18p deletion syndrome are rare. There are no prenatal diagnosis cases of 18p deletion syndrome found by NIPT reported previously. A case of de novo 18p inv-dup-del in a Chinese pregnant woman but not her feus was accidentally discovered by the NIPT during her prenatal examination [25]. Our case indicated that NIPT was also useful as a clue to the chromosome microdeletions and microduplications.

In summary, we present the first case of prenatal diagnosis of 18p deletion syndrome following the NIPT. This report shows that NIPT can give clue to chromosome microdeletions. Further expansion of NIPT through deeper sequencing has focused on additional screening for microdeletion and microduplications. NIPT, combined with ultrasound may be a relatively comprehensive screening strategy for fetal 18p deletion syndrome.

## Data Availability

All data generated or analyzed during this study are included in the published article.

## Abbreviations

aCGH: Array-based comparative genomic hybridization
CMA: Chromosome microarray assay
CNV-Seq: copy number variation sequencing
FISH: Fluorescent in situ hybridization
HPE: Holoprosencephaly
INT: Increased nuchal translucency
NA: Not available (absent or unrecorded)
NIPT: Noninvasive prenatal testing
PPV: Positive predictive value
qf-PCR: Quantitative fluorescent polymerase chain reaction
